# Biomimetic materials assembled on a photovoltaic cell as a novel biosensing approach to cancer biomarker detection

**DOI:** 10.1038/s41598-018-27884-2

**Published:** 2018-07-05

**Authors:** Felismina T. C. Moreira, Liliana A. A. N. A. Truta, M. Goreti F. Sales

**Affiliations:** BioMark-CEB/ISEP, School of Engineering of the Polytechnique Institute of Porto, Porto, Portugal

## Abstract

This work describes for the first time the integration of Dye Sensitized Solar Cell (DSSC) technology in biosensors and biomimetic materials, opening doors towards a new dimension of autonomous screening devices that may be used in point-of-care, with zero-power requirements. DSSCs are fabricated with a counter electrode (CE) of polypyrrole (PPy) that was made responsive to a specific protein by biomimetic material (BM) technology. Carcinogenic embryonic antigen (CEA) was selected as target protein. The resulting BM-PPy film acted as biomimetic artificial antibody for CEA. Rebinding of CEA into this film changed its intrinsic electrical properties and the subsequent electrical output of the DSSC using it as CE. The quantity of CEA in solution was deduced by *I-V* and electrochemical impedance spesctroscopy (EIS). Linear responses to CEA were observed down to 0.25 pg/mL, with 0.13 pg/mL detection limit. Control films of PPy (prepared without CEA in the electropolymerization step) confirmed the ability of the BM material to recognize the target protein. Accurate results were obtained in the analysis of urine samples. Further developments into this ground-breaking self-powered biosensor will display a huge impact in point-to-care medical applications, which may be extended to other fields of knowledge.

## Introduction

Biosensors are an expanding field of (nano)technology^1,2^. These devices moved chemical, biochemical and biological analyses from the conventional laboratory into the field. The most successful commercial biosensor within time remains the glucose meter, combining an electrical reading box with a glucose strip, which is indeed an electrochemical biosensor. The great success of this approach has attracted the attention of researchers over time, driven to explore and develop other electrochemical biosensors, sensitive to other biomolecules of interest in many fields. Electrochemical biosensors combine in a single device a (bio)recognition element (i) and an electrical transducing element (ii).

The biorecognition element (i) is the one responsible for the selectivity of the biosensor (its ability to discriminate a target compound among several others). There are many (bio)recognition elements that may be used for this purpose. The use of biological elements is widely established due to its simplicity and high efficiency. There are however drawbacks: low stability, complex production approaches, high cost and unavailability of some biological targets. Thus, the artificial counterparts have emerged to overcome these disadvantages. Mostly inspired in nature, these materials are robust, mostly stable in harsh conditions and inexpensive^3–5^.

Among synthetic (bio)recognition elements, the use of biomimetic material (BM), also known as plastic antibodies, is increasing^2^. In these, polymeric structures are grown in the presence of a given molecule and this molecule is removed later, to generate vacant positions to which it may rebind with high affinity. The possibility of assembling the polymeric network by means of electrical stimuli has brought several advantages over the conventional polymerisation approaches, highlighting the potential of electropolymerization^6,7^.

The transducing element (ii) is responsible to translate the electrical changes (mostly current, resistance and/or voltage) generated by the localized interaction between the (bio)recognition element and the target compound into a measurable event. In brief, the transducing element sends the electrical change occurring on the biosensor via an electrical signal arriving to the external reading apparatus. The electrical connection between the biosensor and the external apparatus may require electrical cables or may be simplified by the use of printed conductive paths on the biosensor, matching the electrical connections on the reading apparatus (similar to a pen-drive reading in a computer). The size of the apparatus depends mostly on their ability to process more or less complex electrical data and in some cases may require access to an external computer. Overall, the apparatus remains a limiting factor for the miniaturization of the complete set-up, also increasing costs to the final product.

Thus, this work is a first step towards the possibility of monitoring the electrical readings in electrochemical biosensors with a stable and low cost biorecognition element and without needing neither an electrical reading box nor an electrical power source. This is a disruptive approach compared to any existing biosensor, supported by a Starting Grant of the European Research Council. First, an autonomous electrical power is necessary to support electrical autonomy. Thus, electrical independency of biosensors may be achieved by merging photovoltaics and biosensing technology into a single device, an approach never taken before.

In a simple perspective, photovoltaic cells convert solar energy into electrical energy. Many different approaches are employed in the literature for this purpose. Dye-sensitized solar cells (DSSCs) are leading the way, regarding low-cost, high conversion efficiency and easy of fabrication^8,9^. A typical DSSC contains two electrodes and a redox electrolyte system. One electrode is a photoanode electrode, consisting of a dye that absorbs photons (light) and becomes photoexcited, moving electrons up to a higher energy level, and a semiconductor with a porous network (typically of TiO_2_) by which such excited electrons may reach the other electrode through an external circuit. The other electrode is a counter electrode (CE) that receives the electrons flowing from the semiconductor of the photoanode and transfers these into the redox electrolyte. The redox electrolyte is an ion-transporting electrolyte (usually a redox couple 3I^−^/I^3−^) that receives the electrons from the CE (holding catalytic properties against the redox couple) and gives these back to the dye, thereby regenerating it and resetting the system to the original stage.

In terms of configuration, the biosensor could exist in any of the two electrodes of the DSSC and the photovoltaic cell would become the transducer element. Once the target compound binds to that electrode, the electrical performance of the cell changes, expectably in a concentration dependent format. In a first approach, the CE was selected herein. There are several materials employed to generate the CE of DSSC, including, platinum (classical approach), carbon-based materials^10^ (carbon black^11^, graphite^12^, mesoporous carbon^13^, carbon nanotubes^14^) and conducting polymers including poly(3,4-ethylenedioxythiophene)^15^, polyaniline^16,17^ or Polypyrrole (PPy)^18,19^. Teh last one allows easy synthesis, which includes electropolymerization^20,21^, while displaying high catalytic activity towards and iodide redox electrolyte, great stability, and biocompatibility^22–24^.

**Figure 1:**
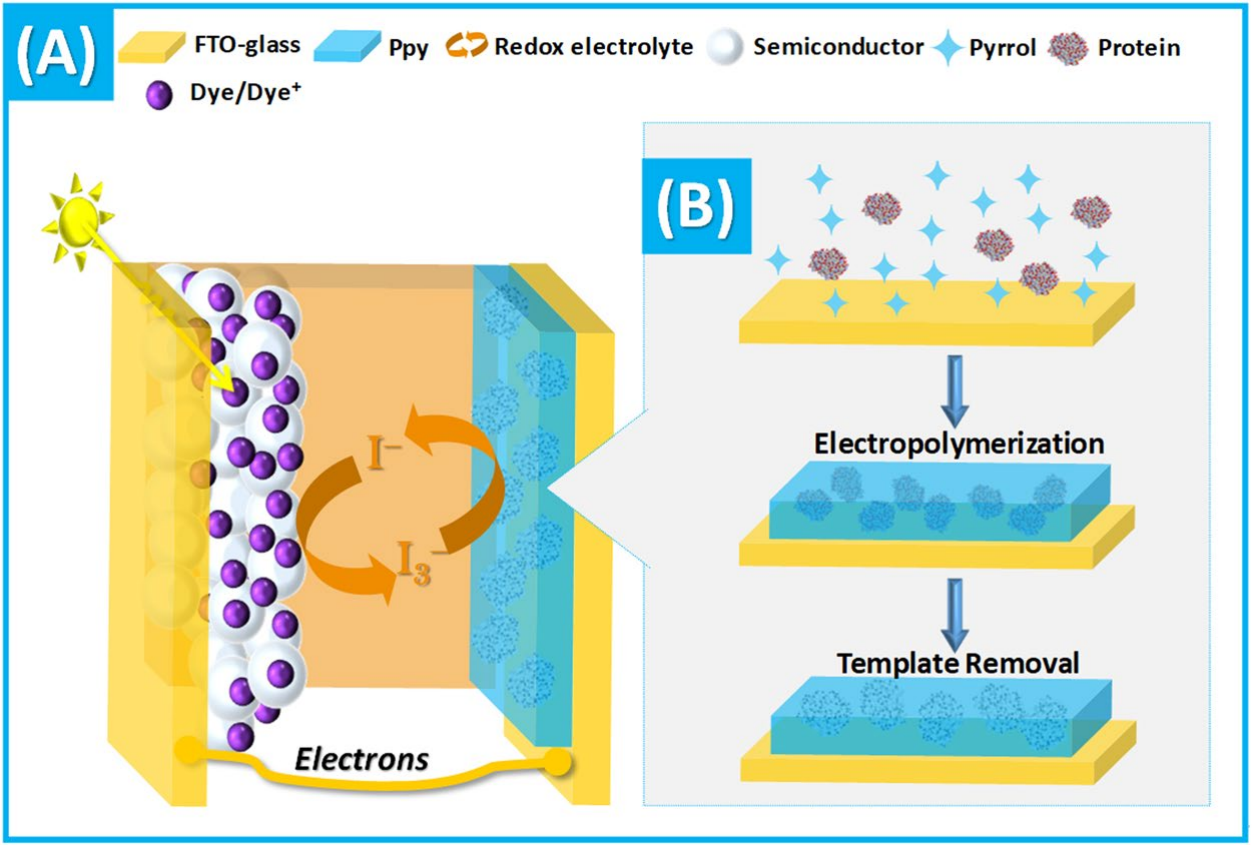
Schematic representation of the Dye Sensitized Solar Cell merged with an imprinted layer of PPy, assembled by electropolymerization.

Thus, this research work is the first one presenting a combination of a biosensing element that is a biomimetic material in a photovoltaic cell. DSSCs were assembled using a dye sensitized TiO_2_ photo-anode and a CE of PPy, obtained by electro-polymerization under BM technology. For this, the polymerization of Py was conducted in the presence of a target analyte (Carcinoembryonic antigen, CEA, a glycoprotein acting as cancer biomarker, selected for this first test) and followed by subsequent removal of that analyte (by enzymatic digestion). The CE acted as a biomimetic material (for CEA) and the photovoltaic cell acted as the electrical reading box (Fig. 1). It was clear that the efficiency of the cell was concentration dependent, yielding an innovative way to monitor the electrical performance of any electrochemical biosensor.

## Results and Discussion

### Production of the sensing polymer

The overall process for producing the sensing polymer is shown in Fig. 1B. It consisted in an imprinting stage where a thin-film of polymeric Py was produced in the presence of CEA and the biomimetic material was formed by removal of the CEA entrapped within the polymeric network.

In general, the electrochemical polymerization of Py involved the oxidation of Py in order to generate a radical cation, occurring when a suitable anodic potential or current was applied to a conducting substrate. These radical cations reacted next with each other to form a radical dimer, which in turn was transformed in a trimer and longer chain lengths^25,26^. In general, Py oxidises between +0.65 V and 0.90 V vs. SCE, but at higher potentials overoxidation of the deposited PPy film occurs, which is an irreversible process that leads to decreased conductivity^27^. Herein, the electro-polymerization of Py was conducted by CV consecutive cycles, between −0.2 and +0.8 V (Figure S1). Within this range, Py displayed electroactive features.

Moreover, the nanostructural features of PPy depend on several parameters used along the electropolymerization process. These parameters include: i) the nature of the electrode^28^; ii) the concentration and nature of the electrolyte^26^; ii) the solvent^29^; iv) the applied potential or current density^30^; iv) the reaction temperature^31^; v) pH; and vi) the mode of electro-polymerization. These factors can affect the rate of polymer growth and its nanostructural arrangement, and determine the type of dopants that will be integrated into the polymer during electro-polymerization, which in turn influence the redox properties and the porosity of the film^26^. Regarding these factors, a PBS buffer of pH 7.2 was used in the electropolymerization process, as the target and final idea was to rebind CEA from biological fluids. The scan-rate was set to 50 mV/s, as our previous BM experiments with Py for other proteins used similar scan-rates.

It is also relevant to mention that PPy displays conductive features, which is a relevant property for merging a biosensor in a DSSC. These conductive properties were evidenced in the CV response (Figure S1), reflected in the significant current increase and in the peak shift to lower potential values. Accordingly, the first cycle showed low current values below +0.65 V, while above this potential the current increased considerably; the polymer started depositing on the conductive glass for potential values above +0.65 V. In the 10^th^ cycle, the polymer started depositing on the substrate at +0.55 V and in the 15^th^ at about +0.45 V. This negative potential shift also confirmed the conductive features of the so formed PPy.

As the polymer was growing by electrical stimuli, CEA was becoming entrapped within the polymeric network. This justified the fact that the growth of the BM-PPy was more limited than the control material, CM-PPy, translated by the lower currents of the imprinted material (Figure S1). The subsequent removal of CEA, created vacant positions on the polymeric film surface. These positions were expected to hold stereochemical and electrostatic complementary features to other CEA proteins, in a similar way to the interaction between antibody and antigen in nature. These vacant sites were therefore sites displaying high affinity for CEA, being also recognized as plastic antibodies. Herein, CEA was removed from the polymeric network by an unspecific protease. Proteinase K was selected for this purpose, for being a highly active and stable protease with low cutting specificity.

### Surface modification Follow-up

All stages involved in the assembly of the sensing polymers were followed by electrochemical and physical characterization. The negative control material obtained by excluding the protein from the polymerization stage, NI-PPy, was characterized in parallel.

### Electrochemical measurements

**Figure 2:**
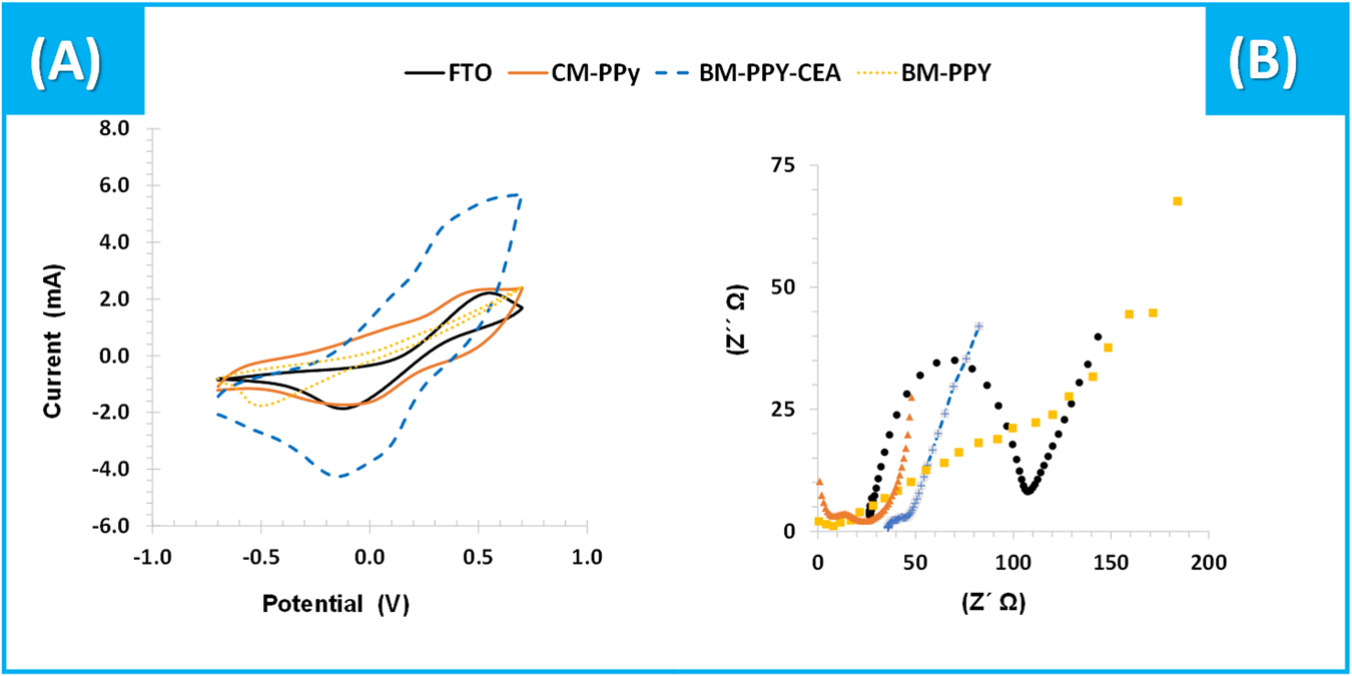
Electrochemical CV (**A**) and EIS (**B**) data of the different steps involved in the assembly of the sensing polymers. Data corresponding to 5.0 mM [Fe(CN)_6_]^3−^ and 5.0 mM [Fe(CN)_6_]^4−^ readings in PBS buffer, pH 7.4.

CV assays are shown in (Fig. 2A) and correspond to the reading of an iron standard redox probe in a three-electrode system, in which the working electrode was the clean FTO-glass, or the BM-PPy films with CEA or a control film CM-PPy. As a conductive support, FTO-glass allowed the detection of both oxidation and reduction peaks of the iron probe. However, its ability to catalyze the oxidation or reduction of iron was small, reflected in the large potential peak separation. The subsequent polymerization of PPy on this support in the presence of CEA, increased significantly the peak currents and reduced the potential peak separation. After template removal, a decrease of the peak current and an increase of the peak separation were observed. It could be related to the presence of the imprinted cavities, which limit the movement of the electrons through the aromatic structure. In addition, a direct contact of the redox probe with the FTO is enabled, due the imprinted cavities on the sensor surface.

In general, EIS data obtained with the BM-PPy material on FTO (FTO/BM-PPy) was consistent with the formation of a conducting layer on the FTO glass (Fig. 2B). The equivalent circuit was used to describe the electrochemical proprieties at the electrode-solution interface, with the occurrence of *Faradaic* current diffusion transport^32^. It consisted in a semicircle and its diameter matched the charge-transfer resistance (Rct)^33^. The Nyquist plots obtained in EIS assays showed that the polymerization of Py on FTO yielded a significant decrease in the Rct (~70 down to ~10 Ω). This observation supported the fact that the PPy film increased the electroactive surface area, resulting in an easier electron transfer from the medium to the electrode. This was also coupled to a slight OCP increase.

In general, the electropolymerization of Py was similar in the presence (BM) and in the absence (CM) of CEA, considering CV data. Overall, the current increased continuously in subsequent cycles (Figure S1). Compared to BM-PPy, the BM-PPy film displayed CV peaks of higher currents (Fig. 2A). This was not obviously consistent with the CV data of the electropolymerized films. However, this could be justified by a more compact PPy film in CM materials, due to the increased polymerization efficiency and to the increased porosity of the BM film in result of the presence of CEA. Thus, the CM film compactness could hinder the diffusion of redox active species, thereby decreasing the corresponding peak currents.

Finally, after CEA removal by an unspecific enzyme, the Rct increased significantly, from ~10 up to ~150 Ω. This could be related to the presence of cavities in the polymeric matrix that allowed a direct access of the iron redox probe to the FTO glass, a material of lower electrocatalytic properties than PPy.

### Fourier Transformed Infrared spectra

**Figure 3:**
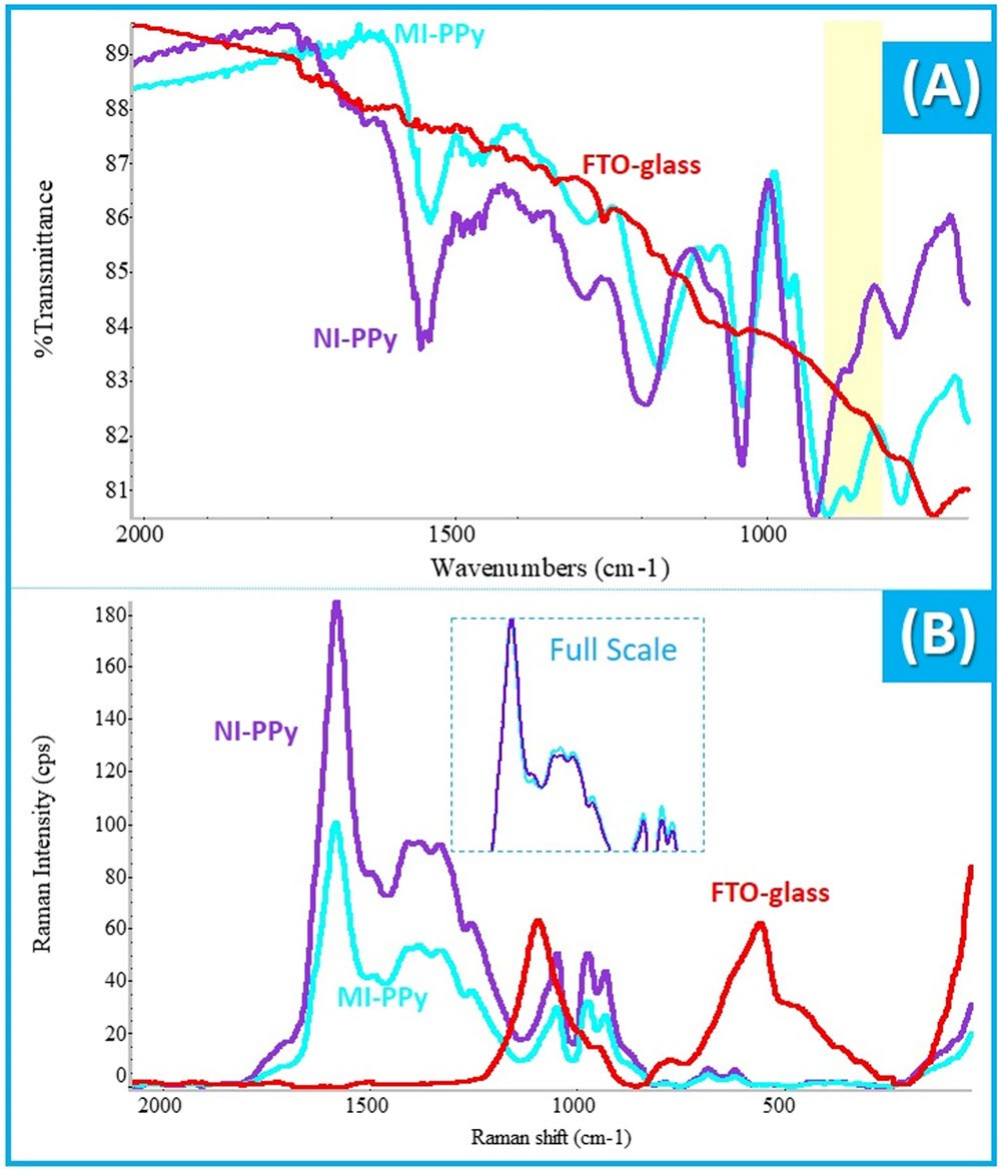
FTIR (**A**) and Raman (**B**) spectra of FTO glass and BM-PPy and CM-PPy films assembled on the FTO glass.

The Fourier Transformed Infrared (FTIR) analysis was conducted directly over the observed materials and the corresponding spectra are shown in Fig. 3A. Overall, the FTO glass had no peak over the observed wavenumber range, meaning that the subsequent peaks detected on the BM-PPy or CM-PPy films accounted solely the presence of PPy + CEA or PPy, respectively.

The peaks observed in BM-PPy, BM-PPy-CEA and CM-PPy materials are listed in Table S1. From these, some peaks may be correlated to the presence of PPy on the FTO glass, while others are related to the fingerprint behavior PPy. The bands at ~1540 cm^−1^ were related to the vibrational stretch of C bonding in the Py ring. The peaks at 1193/1170 and 1039/1037 cm^−1^ were linked to the vibrational stretch of C-H and C-N bonding. The maximum adsorption bands at 900 or 922 cm^−1^ were attributed to the vibrational deformation of the C=C bonding in the aromatic ring of Py. Finally, the adsorption bands at 787 or 783 cm^−1^ were assigned to deformation, in and out of the plane, of C-H bonding in the units of Py.

Overall, the differences between the spectra of BM-PPy and CM-PPy materials are confined to two significant wavenumber peak shifts (Table S1). One was located at 922 cm^−1^ in the CM-PPy, shifting to 900 cm^−1^ in the BM-PPy film. The other was located at 1193 cm^−1^ in the CM-PPy, shifting to 1170 cm^−1^ in the BM-PPy film. This was likely assigned to the presence of CEA within the polymerization stage or to the presence of CEA residues within the polymeric network after protease digestion once these same shift peaks are present in the BM-PPy-CEA sensor with larger peak shift.

### Raman Spectroscopy

Raman spectra are shown in Fig. 3B and are generally consistent with literature data^34^. The relevant peaks observed have been listed in Table S2. The highest intensity peak in all spectra was located at ~1578 cm^−1^ Raman shift. This peak revealed the stretching modes of the C=C bonding present in PPy. The band found at ~1380 was assigned to the anti-symmetric stretching of the C-N bonding in PPy and the band at ~1046 was associated to the in-plane vibrational bonding if C-H and symmetrical reflection planes of the ring. The bands observed at ~971 and ~929 cm^−1^ corresponded to the vibrational deformation of the Py ring.

The two picks at ~1578 (G band) cm^−1^ and ~1380 (D mode) are associated to the G and D band. The D-mode typically reflects a disordered sp^3^-carbon structure, while the G-band reflects the ordered sp^2^-carbon organization. Thus, the increase of I_D_/I_G_ ratio revealed the increasing disorder degree existing within in materials. The CM after template removal and CM materials showed quite similar ratios, of 0.54 and 0.50, respectively, revealing that the template removal from the polymeric matrix did not affect the structure of the polymer and was quite effective. In addition, CM material displayed slightly lower defects than the corresponding imprinted sensor, which was related to the inexistence of CEA within the polymeric matrix. When comparing the BM sensor before (I_D_/I_G_ = 0.67) and after template removal, the polymeric matrix reflected higher level of disorder, due the presence of the protein.

No signal of FTO was detected in these, because the region observed had the FTO completely covered with the PPy film.

### Electron microscopy analysis

**Figure 4:**
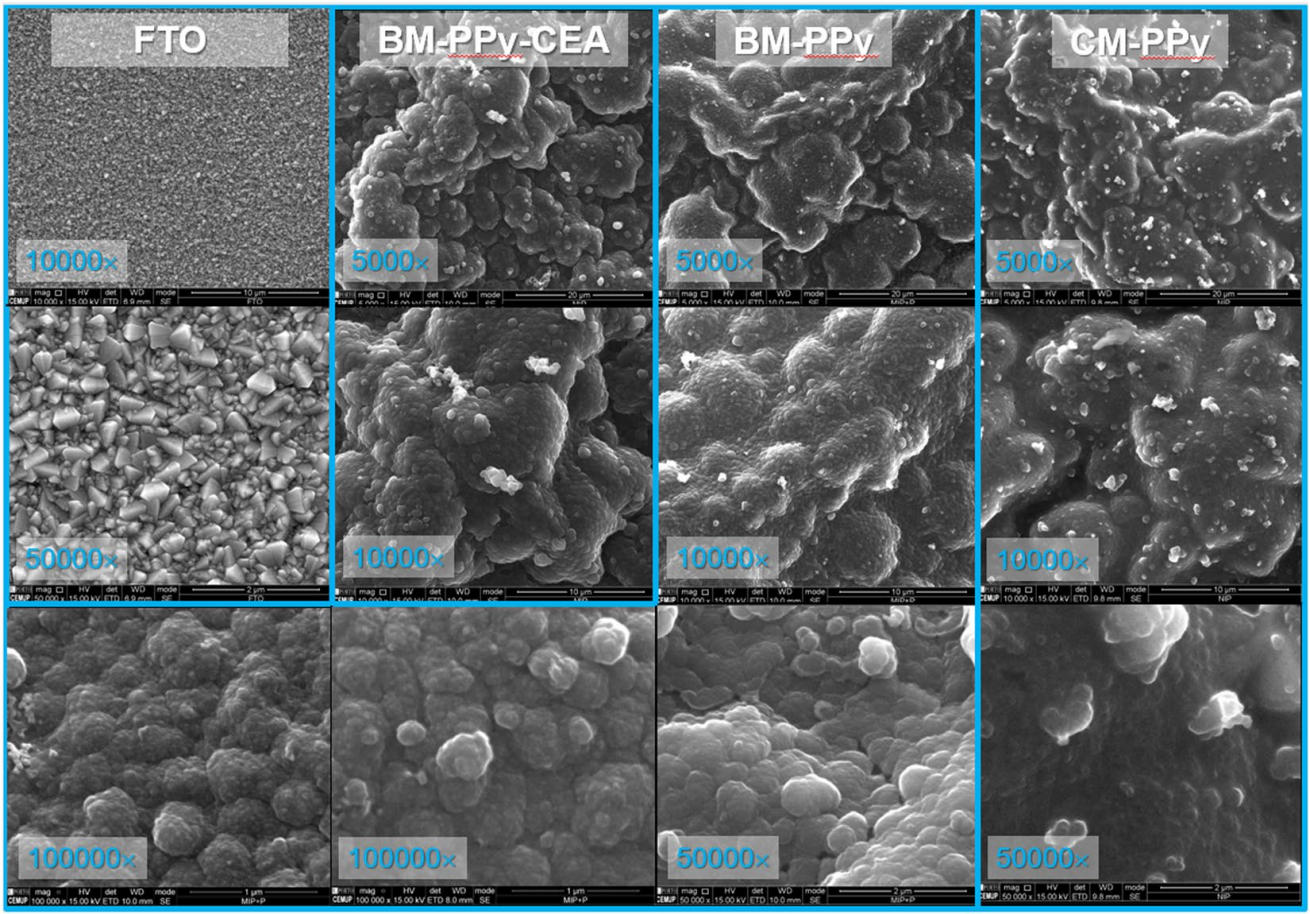
SEM images of clean FTO glass and FTO glass covered with BM-PPy-CEA, BM-PPy or CM-PPy.

Scanning Electron Microscopy (SEM) analysis confirmed the presence of PPy on the FTO glass, as evidenced in Fig. 4. The appearance of the FTO glass surface under 10000× magnification was completely changed, from an apparently flat and granular material to a soft globular structure after PPy formation. Such globular appearance of PPy did not seem to depend from the presence of CEA within the polymeric matrix, nor from its removal.

The presence of CEA within the BM-PPy-CEA film was further suggested by the impossibility to collect a focused SEM image under 50000x magnification: CEA is not a conducting material, and therefore the signal generated was of poorer quality than that of the PPy alone. In the same line of thought, the removal of CEA from BM-PPy-CEA would be confirmed, because the material BM-PPy (obtained by proteinase K treatment of BM-PPy-CEA) gave rise to a SEM image of good quality. In addition, the BM-PPy images showed some pores that were not visible in the CM-PPy, which were assigned to the treatment of proteinase K and subsequent CEA removal. These pores were measured in lower magnification and have ~25 nm size. These materials were further analyzed by EDS (Figure S2), but no valuable information arrived from this data, mostly because the polymeric materials were formed in a complex matrix of PBS.

### Set-up of the DSSC with the biosensor

As explained, a typical DSSC combines three components: (i) a working electrode (WE), consisting of a porous nanostructured TiO_2_ attached to a conducting substrate, often FTO glass, sensitized to light by a light-absorbing dye; (ii) a redox system, containing the redox couple iodide/triiiodide (I^−^/I_3_^−^) in an electrolyte support; (iii) and a CE, that catalysis the electron transfer from the external circuit into the redox electrolyte (Fig. 1).

For the purposes of this work, the CE in the DSSC was replaced by our biosensor, the FTO/BM-PPy film. In terms of performance, the DSSC cell was evaluated as a normal photovoltaic cell, to identify its maximum current, which would correspond to the blank signal; the CE was incubated in buffer before being inserted into the cell. The ability of this set-up to act as an electrical reading box of CEA was further tested by incubating the CE in increasing concentrations of CEA and reading the performance of the photovoltaic cell with this CE previously incubated in CEA.

The set-up was also adapted to handle several open/close movements of the photovoltaic cell, for each sample incubation period, needed along a single calibration. Such open/close requirements to handle the set-up were related only to the calibration procedure, because any sample analysis would require a single reading. One of the effects resulting from the opening of the cell and closing again to read their performance was the loss of the dye adsorbed into the semiconductor layer. A partial loss of the semiconductor material could also occur, although the film seemed integer under naked eye. Thus, the physical integrity of the photoanode electrode was preserved by casting on it a semipermeable hydrophobic and plasticized PVC membrane that allowed ion-exchange. The effect of this layer was tested by evaluating the performance of sequential opening/closing cells. Overall, the use of this membrane endorsed accuracy and reproducibility to the results, although it also introduced an additional resistance into the system, thereby lowering the overall efficiency of the DSSC.

In addition to this, the electrolyte within a typical DSSC used acetonitrile as solvent. This solvent contributed to change the conformation of the protein rebound on the BM layer. Its interaction with the PPy film on the FTO glass was also observed, contributing to its removal from the FTO support. Thus, this solvent was mixed with PBS buffer to reduce this effect. Ionic liquids were kept to ensure electrical conductivity. The consequence of this change was a decrease in the overall cell efficiency, but this was compensated by an increase in the cell stability and reproducibility.

The following procedures are therefore related to the evaluation of blank cells, DSSC/BM-PPy (section 3.4); the evaluation of these cells incubated in increasing concentrations of CEA (DSSC/BM-PPy-CEA), where CEA was present by rebinding from the standard solution (section 3.5); and the subsequent application to the analysis of spiked urine samples (section 3.6).

### Current-voltage characteristics of blank cells, DSSC/BM-PPy

The solar cell performance is typically monitored by its current-voltage *(J-V)* characteristics under specific illumination conditions. The cell was illuminated herein by a calibrated lamp source, consisting of a white LED of 30 W/cm^2^. The current-voltage characteristics were then monitored by varying an external load from zero load (J_sc_, the short circuit current, in mA/cm^2^) to infinite load (V_oc_, open-circuit condition). J_sc_ and V_oc_ corresponded in practice to the maximum current and voltage, respectively, from the cell. In conjunction with J_sc_ and V_oc_, the Fill Factor (FF) determined the maximum power from a solar cell, expressing the “squared shape” of the *J-V* curve; FF was given by equation 1. Moreover, the efficiency of the cell was estimated by the ratio between the power generated by the cell and the power of the incident light (equation 2). In addition, the incident photon-to-current conversion efficiency (IPCE) revealed how efficiently light of a specific wavelength was converted into current (equation 3). IPCE % was defined as the number of electrons generated by the number of photons incident on the photodetector (being *λ* the excitation wavenumber, in nm).

1 $$FF=\frac{{J}_{m {\acute{a}}x}\times {V}_{m{\acute{a}}x}}{{J}_{SC}\times {V}_{OC}}$$

2 $$\eta =\frac{{J}_{SC}\times {V}_{OC}}{{P}_{incident}}$$

3 $$IPCE=\frac{1240\times {J}_{SC}}{\lambda \times Power}$$

The effect of using a BM-PPy film as the CE was tested by comparing a photovoltaic cell assembled with a CE made of FTO glass or FTO glass covered by this film. The IPCE increased from ∼1% to ~5.2% by the presence of the BM-PPy film on the conductive glass. This confirmed the benefits of using a PPy-based CE, displaying a large electrochemical surface area, good film-forming ability and high electrocatalytic activity against the redox probe. The PPy layer also showed lower charge-transfer resistance than the FTO-glass material, which allowed more rapid electron transport and better cell performance.

In agreement with the previous EIS data, when the protein was removed from the PPy network, the IPCE of the cell decreased to ~3.2%. This behavior was assigned to the presence of cavities within the polymeric matrix that allowed a direct access of the probe to the FTO film, thereby reducing the electroactivity properties of the CE.

Overall, the performance of the photovoltaic cell with a CE of BM-PPy film indicated the possibility of using this approach as an electrical reading box of the CEA biosensor. Overall, this innovative approach seemed promising, for creating an innovative and powerful tool for screening cancer biomarkers in point-of-care, and was calibrated after against CEA concentration.

### Calibrations of the DSSC/BM-PPy set-up with CEA standards

**Figure 5:**
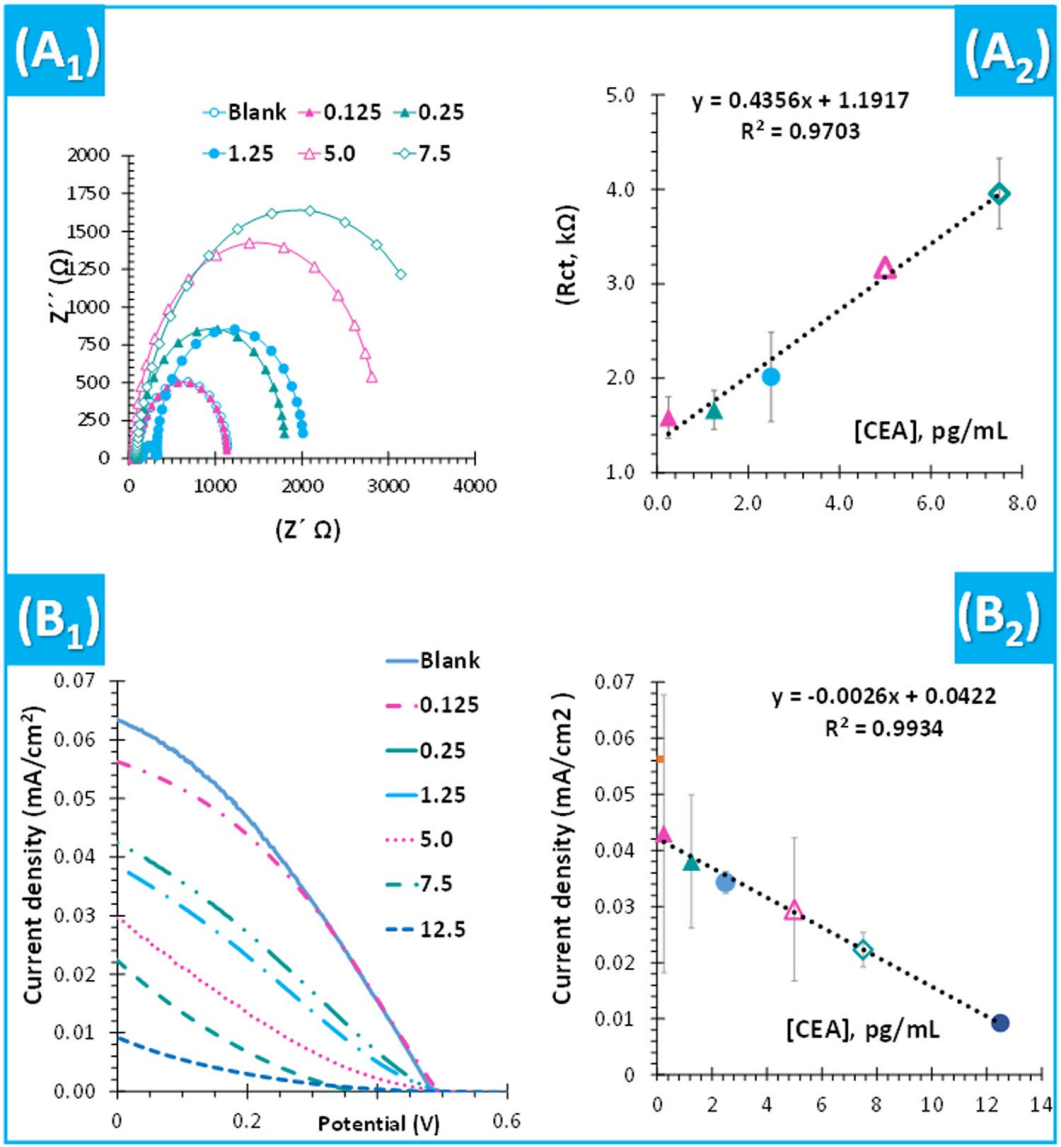
Typical data (1) and representative calibration curves (2) for EIS (**A**) and power (**B**) assays of the DSSC using BM-PPy as CE and an electrolyte of 0.1 M LiI, 0.05 M I_2_, 0.6 M HMII and 0.5 M TBP prepared in PBS. Error bars correspond to 5%.

The analytical performance of the biosensor merged in a photovoltaic cell (DSSC/BM-PPy) was evaluated by calibration curves. For this, the CE was incubated in each standard solution and after set-up in the merged configuration, to evaluate its response in terms of EIS and *I-V* measurements. Calibration curves were recorded for CEs of FTO/BM-PPy (Fig. 5) or the corresponding control material (FTO/CM-PPy). The calibrations plotted current intensity (mA/cm^2^, in *I-V*) or R_ct_ (in EIS) against CEA concentration, ranging from 0.125 to 12.5 pg/mL. This specific concentration range was selected after testing the cell over a wider range of concentrations. In general, the presence of CEA in the redox probe concomitantly decreased the current density, while increasing the resistance of the CE.

Regarding EIS data and comparing to the biosensor in a non-merged configuration, the Warburg’s impedance was no longer present (Fig. 5A), because the diffusion limited behavior was missing in the Nyquist plot. Thus, the circuit consisted only of one resistor (solution resistance, Rs) in series with two parallel circuit with a resistor (Rct and a double layer capacitance, Cdl). In general, the Rct values of the DSSC/BM-PPy configuration increased linearly with the increasing CEA concentration, from 0.125 pg/mL (Fig. 5A). The BM-PPy showed linear responses up to 7.5 pg/mL, with a slope average of 0.436 Ω×mL/pg and squared correlation coefficients >0.97. The LOD was unknown and lower than 0.125 pg/mL.

In *I-V* assays (Fig. 5B), the BM-PPy sensor showed linear behavior down to 0.25 pg/mL, with a slope of −0.0026 (mL×cm^2^)/(mA/pg) and squared correlation coefficients >0.999 and the LOD was less 0.125 pg/mL. The repeatability of the analytical response was good, with standard deviations below 10%. Comparing to EIS, I-V measurements showed a linear response of better quality and over a wider concentration range.

In the CM-PPy sensor, the Rct tended to increase for increasing CEA concentrations, but only after 0.25 pg/mL. Its response was random, with maximum squared correlation coefficients of ~0.96. As in EIS data, the current density of the CM-PPy sensor decreased only after 0.25 pg/mL, also displaying a random behavior (maximum squared correlation coefficients were ~0.662).

Overall, *I-V* data showed quicker, more sensitive and more reliable responses to CEA, when compared to EIS assays. The BM and CM differentiated responses evidenced the ability of the BM-PPy polymer to interact with CEA mostly by its imprinted sites. The CM-PPy response was typically random and mostly assigned to non-specific interactions. In turn, non-specific adsorption in BM-PPy played a minor contribution for CEA recognition.

### Application of the DSSC/BM-PPy set-up to the analysis of urine samples

**Figure 6:**
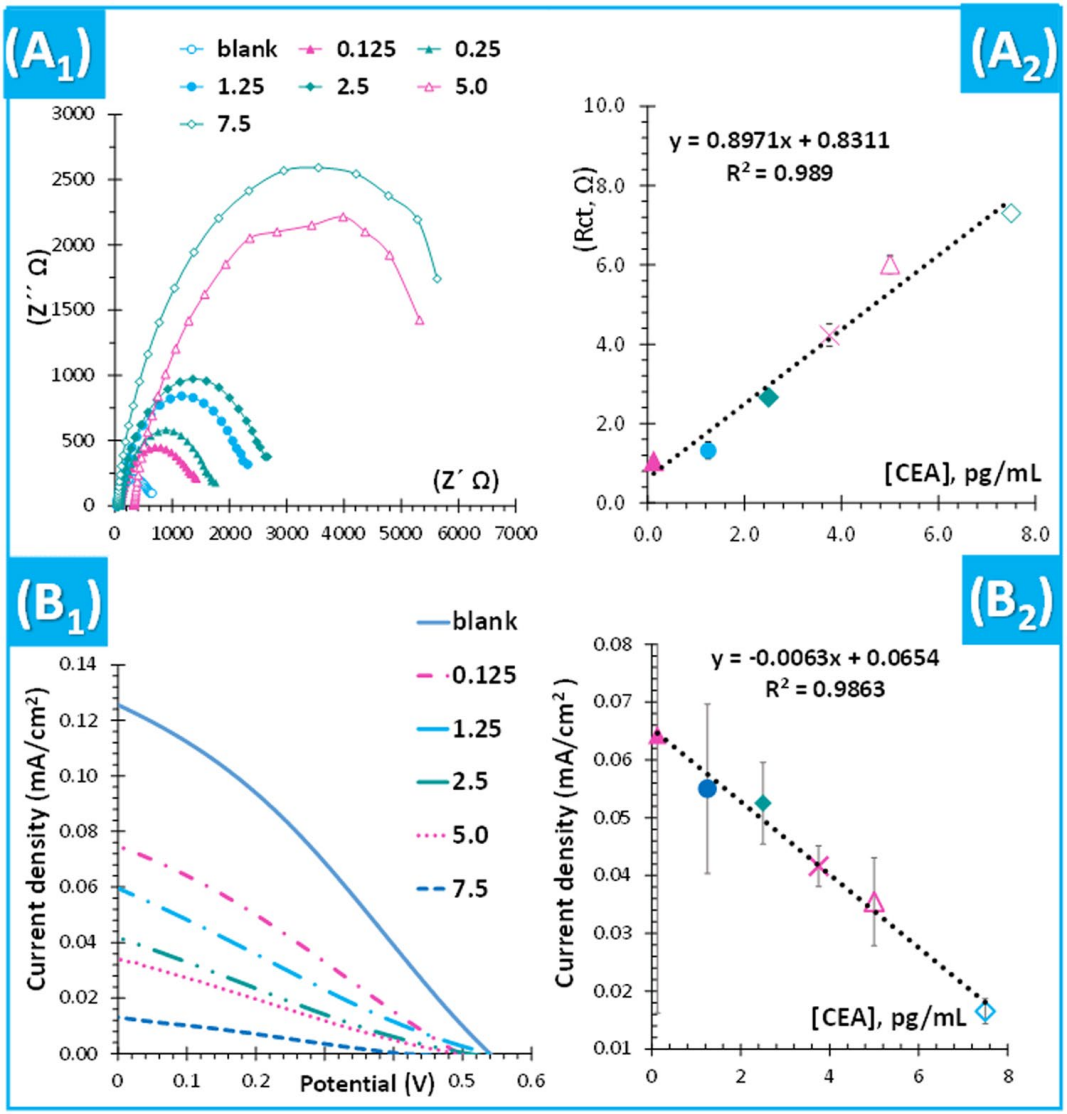
Typical data (1) and representative calibration curves (2) for EIS (**A**) and power (**B**) assays of the DSSC using BM-PPy as CE and an electrolyte of 0.1 M LiI, 0.05 M I_2_, 0.6 M HMII and 0.5 M TBP prepared in diluted urine. Error bars correspond to 5%.

The possibility of using the merged device in the analysis of biological samples was tested herein. Urine samples were selected for this purpose, analysed by EIS and *I-V* assays. A background of samples and standards was matched by preparing standard solutions in diluted urine, coming from a pool of healthy individuals. The resulting calibration data is shown in Fig. 6.

In general, spiked urine samples showed good analytical features. In EIS, the minimum concentration of the linear concentration range was 0.125 pg/mL, the LOD was 0.0832 pg/mL and the slope was ~0.89 Ω × mL/pg. In *I-V* measurements, the minimum concentration of the linear concentration range was 0.125 pg/mL, the LOD was 0.091 pg/mL and the slope was −0.0063 (mL × cm^2^)/(mA/pg).

Comparing with the previous calibrations in PBS buffer, the sensitivity increased in urine-based solutions. This is most likely related to the higher ionic strength of urine samples. These results pointed out the possibility of using the proposed set-up in the analysis of urine samples, if the background of the standard solutions is made of a blank sample (from healthy individuals and without the target analyte).

### Application of the DSSC/BM-PPy in CEA Assay

The analysis of spiked real urine was performed to test a potential application for these devices. Each analysis was made immediately after calibration of the device in urine. The analytical application of the biosensor was tested for BM-PPy (Table S3) and the spiked concentration of CEA were 3.75, 5.0 and 7.5 pg/mL. The recovery ranged from 88.4% to 92.5%. The relative error corresponding to this study varied between 3.8 and 11.6%. The results confirmed that the biosensor is appropriate for application in a real urine samples, where several possible interfering species are present.

## Conclusions

To our knowledge, the use of a photovoltacic cell as an electrical reading system for the detection of a given biomolecule was demonstrated herein for the first time. The electrical power was generated by a DSSC, while the biomolecule recognition was ensured by the CE of this cell. The CE was assembled with a BM film of PPy. PPy improved the electrocatalytic properties of this electrode, which in turn compensated the power decrease achieved by the rebinding the target biomolecule. In addition to this, and as already known in BM technology, the biosensor presented herein demonstrates simplicity in designing, short measuring time, and limits of detection of interest for clinical applications.

This very simple integration of a biorecognition element into a DSSC constitutes a promising tool for establishing in a near future a direct sensing of biomolecules by a self-powered system where a photovoltaic cell is the energy source. In addition, the merged set-up presented herein may be applied to almost any biomolecyle of interest, using an appropriate CE. In terms of self-power, the current system demands further improvements, but other works are progressing in this direction with other promising materials, such as polyaniline. The full autonomy of the device is also being established, in another work to be present later.

## Methods

### Apparatus

Electrochemical measurements were conducted in a potentiostat/galvanostat from Metrohm Autolab, PGSTAT302N, with a Frequency Response Analysis (FRA) module, running on NOVA software. DSSC measurements were made in the same equipment, interfaced with a LED driver accessory, also from Metrohm, operating with 700 mA output, for100 mW/cm^2^, and a warm white LED.

FTIR spectroscopy was made in a Nicolet iS10 equipment, from Thermo scientific. Samples were directly analysed in an attenuated total reflectance (ATR) accessory, containing a Germanium crystal. Scans were recorded from 400 to 2000 cm^−1^ wavenumber, collected for 256 times, with a resolution of 8.

Raman spectroscopy studies used a DXR Raman spectroscope from Thermo Scientific, coupled to a confocal microscope and equipped with a 532 nm laser. The signal-to-noise ratio was set for 300 s, hitting the sample with 1 mW laser power by a 25 μm slit aperture.

SEM and EDS analysis was performed using a High resolution (Schottky) Environmental Scanning Electron Microscope with X-Ray Microanalysis and Electron Backscattered Diffraction analysis: Quanta 400 FEG ESEM/EDAX Genesis X4M.

### Reagents and materials

All chemicals were of analytical grade and water was de-ionized or ultrapure Milli-Q laboratory grade. Potassium hexacyanoferrate III (K_3_[Fe(CN)_6_]), Potassium hexacyanoferrate II (K_4_[Fe(CN)_6_]) trihydrate, Sodium acetate anhydrous, Tetrahydrofuran (THF) and Iodine were obtained from Riedel-deHäen; 1-Hexyl-3-methylimidazolium Iodide, Py, 4-*tert*-Butylpyridine, *O*-Nitrophenyloctyl ether (oNPOE), Poly(vinyl chloride) (PVC) of high molecular weight, and Lithium iodide from Sigma; CEA (from human fluids), and proteinase K from Fluka; TiO_2_ (>99.7%, anatase, <25 nm) and *cis*-*Bis*(isothiocyanato)bis(2,2′-bipyridyl-4,4′-dicarboxylato)ruthenium(II) from Aldrich.

A fluoride-doped tin oxide (FTO) glass (sheet resistance 7.0 Ω/sq) was used as the conductive substrate for photoanode electrode and CE. Conductive glass was masked with tape leaving an open area of 4 cm^2^.

### Preparation of the electrodes

Prior to electrode preparation, the FTO-glass was cleaned with ethanol and after by CV electrochemical procedures (cycling between −0.2 and +0.8 V, with a scan-rate of 50 mV/s, in a solution of H_2_SO_4_ 0.5 mol/L).

### Preparation of the photoanode

The semiconductor is prepared by mixing 6.0 g of TiO_2_ powder with 8.0 ml of ethanol, 1.0 ml of acetic acid and 1.0 ml of water. The resulting paste was stirred at room temperature for 1 hour and subsequently applied on the FTO glass by using doctor blade technique^9,35^. The resulting TiO_2_ layer was fired at 450 °C for 1 h. After cooling, the semiconductor film was sensitized to light by immersing it in a solution of 0.3 mM *Cis*-Bis(isothiocyanato)bis(2,2′-bipyridyl)-4,4′-dicarboxilate) Ruthenium(II) dye, prepared in absolute ethanol. The sensitization time was set to 20 h. The electrode was removed after from the dye solution, thoroughly washed with ethanol, and dried.

### Preparation of the counter electrode

The electro-catalytic element of the CE was PPy, prepared *in-situ* via electro-polymerization of Py on clean FTO-glass substrates. BM-PPy films were obtained by CV cycling (30 cycles) in a solution containing Py (0.3 mol/L in PBS buffer) and CEA (0.25 ng/mL). CV scans ranged between −0.2 and +0.8 V, with a scan-rate 50 mV/s. The resulting material (FTO/BM-PPy-CEA) was thoroughly washed with deionized water and incubated overnight in proteinase K (500 µg/mL, prepared in PBS buffer, pH 7.4), in the dark, to remove the protein template. The resulting material (FTO/BM-PPy) should not have CEA at the external surface. It was then washed several times with PBS buffer, to remove peptide fragments and adsorbed proteinase K, and finally rinsed with MQ water. Blank materials were produced in parallel using the same procedure, but without CEA. These materials are usually recognized as biomimetic control (CM) and were assigned as FTO/CM-PPy.

### Physico-Chemical characterization of the films

FTIR, SEM and Raman spectroscopy were used to characterize the films. All materials were analysed directly, as no pre-treating procedures were necessary. Data was collected for FTO glass without any treatment, BM-PPy film with CEA entrapped, BM-PPy film after proteinase K treatment, and CM-PPy films.

### Evaluation of the CEA biosensor in a conventional format

#### Overall set-up

The evaluation of the performance of the CEA biosensor prepared on FTO-glass (which acted after as the CE in the DSSC format) was made first in a three electrode conventional system. For this purpose, the electrode was dipped in a solution, together with a reference electrode of AgCl/Ag, double-junction, and a counter electrode made of platinum.

#### Electrical performance

The electrochemical performance of the CEA biosensor was assessed by CV and EIS assays, in a redox couple solution of 5.0 mmol/L of [Fe(CN)_6_]^3−^ and 5.0 mmol/L of [Fe(CN)_6_]^4−^, prepared in PBS buffer, pH 7.4. EIS used an open potential circuit (OCP), with a sinusoidal potential perturbation, and an amplitude of 0.01 V, over a number of frequencies equal to 50 that were logarithmically distributed over a frequency range of 0.1–100 kHz. The resulting Nyquist plots were fitted to the equivalent circuit in Figure S3, using the NOVA Software. CV assays considered scanning from −0.2 to +0.8 V, at a scan-rate of 50 mV/s. All assays were conducted in triplicate.

### Evaluation of the CEA biosensor coupled to the DSSC

#### Overall set-up

The DSSC cell had a conventional set-up, organized as FTO-glass/TiO_2_/dye//electrolyte//CE/FTO-glass, where the CE was BM-PPy or CM-PPy. The electrolyte was prepared with 0.1 M LiI, 0.05 M I_2_, 0.6 M 1-hexyl-3-methyl-imidazolium iodide (HMII) and 0.5 M 4-tert-butyl pyridine (TBP) in acetonitrile. The photoanode electrode and the CE were placed against each other and separated by a thin spacer; this set-up was then placed on the LED Driver support of the Autolab and the redox electrolyte was allowed to flow through the top of the cell, in-between the two electrodes, thereby closing the electrical circuit.

As the photoanode and counter electrodes would be separated in several occasions, a film of plasticized PVC membrane was placed on the photoanode to prevent dye desorption. The inspiration of the PVC membrane came from ion-selective electrodes based on PVC membranes. It was prepared with ~210 mg PVC and ~75 mg *o*NPOE, dispersed in ~3.5 mL THF, mixed and stirred until the PVC was well moistened. A volume of about 200 μL of this solution was casted on the photoanode and let dry for 24 h.

#### Electrical performance

The *I–V* response of the photovoltaic cell used electrochemical measurements, under 43.0 mW/cm^2^ illumination. EIS analysis was performed in the same equipment, by using the FRA module in a frequency range 0.1–105 Hz. The cell area was always 4 cm^2^.

#### Analysis of spiked real urine samples

The analysis of the real spiked urine samples samples were performed by LSV measurements. The concentration of CEA was ranged between 3.5 and 7.5 ng/pg and diluted in urine 2 times diluted.

#### Calibration of the biosensors

Calibration curves were performed by EIS or EIS/I-V measurements. EIS tested the conventional biosensor format, in which electrical alterations were evaluated by reading the standard iron redox probe. EIS/I-V tested the DSSC-merged biosensor format, in which the DSSC cell contained a CE of BM-PPy, and the data was collected for an iodide-based redox electrolyte. Before starting the calibration of FTO/BM-PPy or FTO/CM-PPy material, a blank solution (buffer or urine sample) was incubated in the polymeric film, for a given time, until a stable electrical reading was reached.

Calibrations started by incubating the CEA standard solution of lower concentration in the polymeric film, for a given time (the same time as with the buffer solution); after this, the polymeric film was washed and the redox probe read, in agreement with the setup under evaluation. This procedure was repeated for each concentration of CEA, tested from the lower to the higher level. CEA concentrations ranged from 0.125 to 12.5 pg/mL, in PBS buffer, pH 7.4.

Calibration plots displayed the electrical signal response against concentration and were evaluated with regard to the linear regression obtained. The limit of detection (LOD) was calculated by the formula 3σ/*S*, where *S* was the slope of the linear calibration plot, and σ the standard deviation of the blank solution (buffer or blank sample).

The possibility of testing the biosensors in biological fluids was evaluated for urine samples. For this purpose, urine samples from health individuals were collected (after informed consent) and spiked with CEA. Prior to analysis, urine samples were diluted 2 times, in a concentration range between 0.125 and 12.5 pg/mL. In general, data reported herein corresponded to a minimum of 3 evaluations.

## Electronic supplementary material

Supplementary information
