# Supplementary material for: Biomimetic materials assembled on a photovoltaic cell as a novel biosensing approach to cancer biomarker detection
Source: Sci Rep. 2018 Jul 5;8:10205. doi: 10.1038/s41598-018-27884-2 (PMC6033912; doi:10.1038/s41598-018-27884-2)
Supplement: Supplementary file 1 — Supplementary information [file 41598_2018_27884_MOESM1_ESM.docx]

**Supplementary Material**

**Biomimetic materials assembled on a photovoltaic cell as a novel biosensing approach to cancer biomarker detection**

*Felismina T.C. Moreira^1)^, Liliana A.A.N.A Truta^1)^, M. Goreti F. Sales^*^*

BioMark-CEB/ISEP, School of Engineering of the Polytechnique Institute of Porto, Portugal

**Figure S1 –** Electrosynthesis of BM-PPy and CM-PPy materials on a clean FTO-glass support (consecutive cycles correspond to increasing currents).

**Figure S2 –** EDS plots corresponding to the SEM images in the main article (Figure 3).

**Figure S3 –**Equivalent circuit model parameters used for fitting the EIS representative of the DSSC/BM-PPy sensor.

(Figure 3).

**Table S1**

List of FTIR peaks and corresponding intensities of the CM-PPy and BM-PPy materials, along with the wavenumber shifts.

| **Wavenumber** (cm^-1^) | | **Transmittance (%)** | | **Wavenumber shift**  (cm^-1^) |
| --- | --- | --- | --- | --- |
| *CM-PPy* | *BM-PPy* | *CM-PPy* | *BM-PPy* |  |
| 787.51 | 783.17 | 96.338 | 68.161 | −4.3 |
| 867.09 | 863.90 | 96.098 | 68.321 | −3.2 |
| 922.44 | 900.44 | 95.101 | 67.635 | −22.0 |
| 963.62 | 963.63 | 96.270 | 76.769 | +0.01 |
| 1038.77 | 1036.94 | 95.451 | 72.217 | −1.8 |
| 1086.77 | 1092.15 | 96.664 | 78.321 | +5.4 |
| 1193.05 | 1170.18 | 95.873 | 73.745 | −22.9 |
| 1288.20 | 1287.55 | 96.606 | 79.845 | −0.7 |
| 1541.24 | 1539.58 | 96.315 | 79.923 | −1.7 |
| 1554.68 | ⎯ | 96.258 | ⎯ | ⎯ |

**Table S2**

List of Raman Shift values peaks and corresponding Raman intensities of the CM-PPy and BM-PPy materials, along with the differences between Raman shifts.

| **Raman Shift (cm^-1^)** | | **Intensity (cps)** | | **Δ Raman Shift**  (cm^-1^) |
| --- | --- | --- | --- | --- |
| *CM-PPy* | *BM-PPy* | *CM-PPy* | *BM-PPy* |  |
| 928.27 | 929.62 | 43.504 | 26.449 | +1.4 |
| 971.84 | 972.18 | 50.391 | 31.988 | +0.3 |
| 1045.98 | 1046.68 | 49.948 | 29.473 | +0.7 |
| 1377.70 | 1381.78 | 92.796 | 53.158 | +4.1 |
| 1578.48 | 1580.42 | 184.755 | 100.331 | +1.9 |
